# Downstream mediators of Ten-m3 signalling in the developing visual pathway

**DOI:** 10.1186/s12868-017-0397-5

**Published:** 2017-12-06

**Authors:** Kelly A. Glendining, Sam C. Liu, Marvin Nguyen, Nuwan Dharmaratne, Rajini Nagarajah, Miguel A. Iglesias, Atomu Sawatari, Catherine A. Leamey

**Affiliations:** Discipline of Physiology, School of Medical Sciences and Bosch Institute, F13, University of Sydney, Sydney, NSW 2006 Australia

**Keywords:** Teneurin/Odz, Binocular vision, EphA7, Zic2, Retinotopic mapping, Neural development, Ipsilateral, Contralateral, Retina

## Abstract

**Background:**

The formation of visuotopically-aligned projections in the brain is required for the generation of functional binocular circuits. The mechanisms which underlie this process are unknown. Ten-m3 is expressed in a broad high-ventral to low-dorsal gradient across the retina and in topographically-corresponding gradients in primary visual centres. Deletion of Ten-m3 causes profound disruption of binocular visual alignment and function. Surprisingly, one of the most apparent neuroanatomical changes—dramatic mismapping of ipsilateral, but not contralateral, retinal axons along the representation of the nasotemporal retinal axis—does not correlate well with Ten-m3’s expression pattern, raising questions regarding mechanism. The aim of this study was to further our understanding of the molecular interactions which enable the formation of functional binocular visual circuits.

**Methods:**

Anterograde tracing, gene expression studies and protein pull-down experiments were performed. Statistical significance was tested using a Kolmogorov–Smirnov test, pairwise-fixed random reallocation tests and univariate ANOVAs.

**Results:**

We show that the ipsilateral retinal axons in Ten-m3 knockout mice are mismapped as a consequence of early axonal guidance defects. The aberrant invasion of the ventral-most region of the dorsal lateral geniculate nucleus by ipsilateral retinal axons in Ten-m3 knockouts suggested changes in the expression of other axonal guidance molecules, particularly members of the EphA–ephrinA family. We identified a consistent down-regulation of *EphA7*, but none of the other EphA–ephrinA genes tested, as well as an up-regulation of ipsilateral-determinants *Zic2* and *EphB1* in visual structures. We also found that Zic2 binds specifically to the intracellular domain of Ten-m3 in vitro.

**Conclusion:**

Our findings suggest that Zic2, EphB1 and EphA7 molecules may work as effectors of Ten-m3 signalling, acting together to enable the wiring of functional binocular visual circuits.

**Electronic supplementary material:**

The online version of this article (10.1186/s12868-017-0397-5) contains supplementary material, which is available to authorized users.

## Background

The generation of visuotopically-aligned circuits is fundamental to binocular visual function. Knowledge regarding the molecular mechanisms which specify ipsilateral versus contralateral identity [1, 2] as well as the mapping of contralateral projections [3–5], has increased markedly over recent years. We still, however, know very little about how ipsilateral inputs are visuotopically aligned with their contralateral counterparts in central targets.

The mapping of contralateral retina onto both the superior colliculus (SC) and dorsal lateral geniculate nucleus (dLGN) relies on EphA–ephrinA interactions [3, 4, 6]. This has been best described for the SC where a low-rostral to high-caudal ephrinA gradient interacts in a repellent manner with the high-temporal to low-nasal retinal EphA gradient to help confine the branching of retinal ganglion cell (RGC) axons to topographically appropriate regions [3, 4, 7]. This is thought to prevent retinal axons from branching caudal to their topographically appropriate terminal zone. Conversely, a reverse gradient of high-nasal and low-temporal retinal ephrinA expression is thought to interact with a gradient of high-rostral to low-caudal EphA expression in the SC to inhibit RGC axonal branching rostral to the appropriate termination zone [8]. Similar gradients and interactions are thought to underlie retinogeniculate mapping [6, 9].

Mapping of the dorsoventral retinal axis onto the mediolateral axis of the SC is mediated by interactions between EphB receptors and ephrinB ligands [5]. Other factors such as Wnt–Ryk interactions [10] as well as Bone Morphogenic protein 4 [11] are also thought to contribute to this process.

The mechanisms underlying the formation of binocular maps are less well understood [12]. EphA–ephrinA interactions are involved in both ipsilateral and contralateral mapping [3, 4, 6, 9, 13]. In order for aligned binocular visuotopic maps to form, however, the mapping of the ipsilateral projection must be reversed across the temporonasal retinal axis with respect to contralateral mapping (reviewed in [12]). Our current knowledge of EphA–ephrinA interactions does not explain how the alignment of ipsilateral and contralateral terminals is achieved. One candidate that shows promise with regard to increasing our understanding of this process is Ten-m3, as its deletion differentially affects ipsilateral versus contralateral retinal projections [14].

Ten-m3 is a member of the Ten-m/Odz/Teneurin family of type II transmembrane glycoproteins which play roles in axon guidance [15], as well as synaptic targeting and organisation in invertebrates [16, 17]. Multiple lines of evidence support similar roles in vertebrates [18–21]. Ten-m3 was identified as a potential guidance molecule in the visual pathway of mice [22], and is expressed in a high-ventral to low-dorsal gradient in the retina. This gradient is conserved in a topographically-corresponding manner in the SC and dLGN [14, 23]. In its absence, ipsilateral retinal projections are dramatically miswired, with terminals no longer confined to their usual target areas in the dorsomedial dLGN and rostromedial SC. Contralateral axons, however, display only subtle changes [14, 23]. This predominantly monocular influence has significant deleterious consequences for vision in Ten-m3 knockout (KO) mice [14]. The molecular pathways to which Ten-m3 contributes to regulate ipsilateral mapping have, however, remained elusive.

The topographically corresponding gradients of Ten-m3 across the visual pathway are consistent with the suggestion that Ten-m molecules may interact directly with each other to specify synaptic matching, as postulated in *Drosophila* [16, 17]. The mapping deficits we have observed in the visual pathway of Ten-m3 knockout (KO) mice, however, include dramatic changes across the representation of the temporonasal retinal axis, which is orthogonal to its expression gradient [23]. It is unlikely, therefore, that the effects on mapping are entirely due to direct interactions between Ten-m3 molecules, leading us to hypothesise that there may be other mechanisms by which this protein acts to regulate neural connectivity. Further, while ipsilaterally-projecting population of RGCs is confined to the ventrotemporal retinal crescent [24] Ten-m3 is expressed more broadly [14, 23]) across this region. Thus, Ten-m3’s critical role in ipsilateral, but not contralateral, mapping does not fit easily with its expression pattern.

One means by which Ten-m3 could selectively influence ipsilateral mapping could be via interactions with molecules that are differentially expressed between the RGCs whose axons form the crossed and uncrossed pathways. Multiple members of the Teneurin family have been shown to undergo a form of proteolysis whereby the intracellular domain is cleaved and translocated to the nucleus where it can regulate transcription [25, 26]. Accordingly, Ten-ms can potentially alter the expression of other guidance molecules. While this has not yet been directly demonstrated for Ten-m3, a nuclear localisation signal and a potential cleavage site are both present on its intracellular domain [27, 28], suggesting a similar transcriptional role for this protein. Moreover, the intracellular domain of another Ten-m family member, Ten-m2, has been shown to interact with Zic1; Zics1–3 have all been shown to have an association with the ipsilateral retinal pathway [1, 29], and Zic2 has been shown to play a critical role in the specification of laterality in RGCs [1].

We sought to identify the signalling pathways to which Ten-m3 contributes in order to better understand the molecular basis for the proper formation of binocular visual circuits. We first show that the mapping deficit in Ten-m3 KOs is due to axonal guidance errors which impact the emergence of ipsilateral retinal axons from the optic tract, suggesting the involvement of EphA–ephrinA signalling. A screen for changes in *EphA*–*ephrinA* gene expression in Ten-m3 KOs revealed that *EphA7* is specifically down-regulated in the visual pathway. We also probed for interactions with molecules associated with the ipsilateral retinal pathway [1, 2, 29]. We found that the intracellular domain of Ten-m3 is able to bind to Zic2 in a pull-down assay, and that *Zic2* and *EphB1* mRNA are both up-regulated in Ten-m3 KOs. These observations help to explain the specific but profound influence of Ten-m3 on the formation of binocular visual circuits.

## Methods

All experiments were performed in mice and were approved by the University of Sydney Animal Ethics Committee and in accordance with NHMRC guidelines. Homozygous Ten-m3 KO (−/−) mice were bred from heterozygotes and compared to Ten-m3 (+/+) age-matched wild type (WT) littermate controls [14]. All experiments used KO and WT littermates from at least 2 and up to 5 litters. Animals were housed in standard mouse cages at an ambient temperature of 23.5 °C. Mice were provided with mouse chow and water ad libitum and maintained on a 12/12 h light/dark cycle.

### Anterograde tracing studies

Animals were anesthetised by inhalation of 2–4% isofluorane in oxygen. Three to six animals were used in each age group: postnatal day (P)0, P3, P6, P9. The eye was opened by making an incision along the palpebral fissure and 0.5 μL of 1% cholera-toxin subunit B (CTB, Invitrogen) was injected into the vitreous chamber. Following recovery from anesthesia, pups were returned to their mothers for a survival period of 1 day to allow dye transport, before they were euthanized by an overdose of sodium pentobarbitone (< 100 mg/kg i.p.). Mice were perfused with 0.9% saline followed by 4% paraformaldehyde in 0.1 M phosphate buffer (PB). Tissue was cryoprotected in 30% sucrose in 0.1 M PB, and sectioned coronally at 60 μm on a freezing microtome. Tissues were counterstained with 4′,6-diamidine-2′-phenylindole dihydrochloride (DAPI) and viewed and photographed on a Zeiss epifluorescence deconvolution microscope using appropriate filter sets.

For quantitative analysis, images of 6–8 serial sections through the dLGN from 3 KO and 3 WT animals were used. Images were thresholded, and the pixel counts across the dorsomedial to ventrolateral (DM-VL) axis of the dLGN were divided into percentile bins for each sample. These distributions were summed for each genotype and normalised to the maximum value for each group to give a relative measure. The distributions were compared using a two sample Kolmogorov–Smirnov test (a significance value of α = 0.05 was used).

### Realtime qPCR

Ten-m3 KO or WT mice were euthanized on the day of birth by an overdose of sodium pentobarbitone as above. The retina, SC, and dLGN were dissected and placed in RNA*later* at 4 °C for 0–4 days. Tissue samples were pooled (n = 3–4 mice) and total RNA was extracted using Trizol (Invitrogen) according to the manufacturer’s instructions. cDNA was synthesised from 3 to 4 independent pooled samples per genotype and quantitative real time PCR (qPCR) was performed as described in [22] normalised to *Gapdh* levels. Technical replicates of each sample were run in triplicate from each sample for each gene tested, with the exception of *Zic2* which was run in quadruplicate. Primer sequences were designed using Netprimer (PREMIER Biosoft International) and Primer3 software [30] based on NCBI Reference Sequences for mRNA (primer sequences and accession numbers listed in Table 1). Primer pairs were designed to have minimal secondary structure, an annealing temperature of approximately 60 °C, an amplicon of approximately 200 bp to flank exon boundaries (as documented in NCBI), and to avoid amplification of trace genomic DNA. Primer specificity was established by comparison with known genomes and sequences using the Basic Local Alignment Search Tool (BLAST) program (http://www.ncbi.nlm.nih.gov) with a maximum cut-off of 70% homology with non-target sequences. Desalted oligonucleotides were manufactured by Sigma Genosys.

**Table 1:** Primer sequences forward and reverse primer sequences for real time qPCR and in situ hybridisation analysis of Eph/ephrinA mRNA expression in visual structures of P0 *Ten*-*m3* KO mouse

Gene name	NCBI mRNA accession #	Primer sequences 5′–3′
Glyceraldehyde-3-phosphate dehydrogenase (*Gapdh*)	NM_001001303	**F_**AACTTTGGCATTGTGGAAGG **R_**ACACATTGGGGGTAGGAACA
EphrinA2 (*Efna2*)	NM_007909	**F_**AGCATCAACGACTACCTGGA **R_**AAAAGGGGGTGAAGAGTTGG
EphrinA5 (*Efna5*)	NM_010109.3	**F_**AGCAACCCCAGATTCCAGAGG **R_**TTTTCCGAGAACTTCAGCG
Eph receptorA5 (*Epha5*)	NM_007937.3	**F_**TGCTATTCGGCACCTCTAAAGG **R_**GGGGGCATAGTTCTCATCAA
Eph receptor A6 (*Epha6*)	NM_007938.2	**F_**CTCCAGCCTTCCCTTCACC **R_**TATCTATCATCAAACTCCACACA
Eph receptor A7 (*Epha7*)	NM_010141.3	**F_**GCTGTAAATGGAGTTTCGGAC **R_**GTGTGGCAACATCAAGCCTA
Zinc finger transcription factor 2 (*Zic2*)	NM_009574.3	**F_**AATGGCTTATTGGCTTATTGG **R_**ACTTTGGCACGGCTCATATT
Ephrin ligand B1 *(EfnB1)*	NM_010110.4	**F_**ACCCGAGCAGTTGACTACCA **R_**ATGATGAGCAGGAAGATGACAC
Eph Receptor B1 *(EphB1)*	NM_173447.3	**F_**TGGCTATGGCAAGTTCAGTG **R_**CAATGTAGATCTTCATCCCT

*Gapdh* was used as a reference gene in real time qPCR assays

Fold change differences between genotype at P0 in EphA/ephrinA expression levels were compared using the Relative Expression Software Tool (REST 2005 BETA V1.9.2) and a pairwise fixed random reallocation test [31, 32]. A significance value of α = 0.05 was assumed.

### In situ hybridisation

Animals were euthanized as described above and decapitated. Heads were frozen in isopentane on dry ice. Cryostat sections, 15 μm thick, were cut and RNA probes were prepared as described in [22]. In situ hybridisation was performed using 200 bp digoxigenin labelled probes as described in [22]. For whole mount in situ hybridisation, animals were euthanized and perfused with 4% paraformaldehyde prior to processing. Reactions were developed using either a fluorescein tyramide signal amplification kit (Perkin-Elmer) or nitroblue tetrazolium chloride and 5-bromo-4-chloro-3-indolyl phosphate substrate (Roche) in detection buffer (100 mM NaCl, 100 mM Tris–HCl pH9, 0.01% Tween20) as per manufacturers’ instructions.

### Ephrin-AP and Eph Receptor-AP affinity probes

EphA/ephrinA binding was examined using EphA3 and ephrinA5 probes conjugated to alkaline phosphatase [33], which exploits the binding promiscuity within the Eph/EphrinA protein family; the EphA3-AP fusion probe binds to ephrinA2 and ephrinA5, and the ephrinA5-AP probe binds all EphA receptors (Eph Nomenclature Committee). Wholemount preparations of SC were lightly fixed in 4% paraformaldehyde before being incubated in the fusion probes. Tissue was rinsed, and endogenous alkaline phosphatase was deactivated by incubation at 65 °C for 3 h to overnight, before reacting to reveal the binding of alkaline-phosphatase tagged probes.

### Microscopy and image analysis

Images of wholemounts were taken on a Wild Heerbrugg M400 light microscope attached to an Olympus DP7 digital camera. Sections were imaged using either a Zeiss Axioplan 2 deconvolution microscope attached to a Zeiss AxioCam HR digital monochrome camera, or an Olympus BX51 microscope attached to a Leica DC500 digital colour camera. Images were viewed using Axiovision 4.7 software (Zeiss).

For in situ hybridisation analysis, the relative intensity of staining was analysed in ImageJ (NIH). Intensity values were sampled from 11 equidistant regions along the rostrocaudal axis of the SC. Values obtained from KO and WT samples were compared using a univariate ANOVA (genotype as between subject factor), followed by Bonferroni corrected pairwise comparisons for each region (a significance value of α = 0.05 was assumed).

### Subcloning of Ten-m3 intracellular domain gene fragment into pGEX expression vector

The intracellular domain of Ten-m3 was sub cloned into a pGEX-6P3 vector (GE Healthcare Life Sciences, Australia) which includes a Glutathione-S-transferase (GST) expression moiety. DNA was sequenced to confirm the orientation and correct reading frame for protein translation. Molecular weight of the fusion protein was estimated using Bioedit based on the amino acid composition and determined to be approximately 58 kDa (Ten-m3 insert ≈ 31.82 kDa; GST ≈ 26 kDa). Growth conditions for protein expression were determined empirically.

Ten-m3-GST and GST alone attached to sepharose beads were incubated with P1 whole brain total cell lysate. Fusion proteins attached to sepharose beads were retrieved by centrifugation, washed and resuspended in 2X Laemmli buffer pH 6.8 (20% glycerol, 4% SDS, 120 mM Tris–HCl, 10% β-mercaptoethanol, 0.02% (w/v) bromophenol blue). A third beads only control sample was prepared by resuspending sepharose beads in PBS.

### SDS-PAGE and Western blot analysis

Proteins separated by SDS-PAGE were transferred to a polyvinylidene fluoride membrane (Millipore). Successful transfer was visually confirmed using Ponceau S staining prior to blocking. Membranes were blocked overnight in blocking buffer (5% skim milk powder in Tris buffer: 2.5 mM Tris, 15 mM Sodium Chloride, 0.005% Tween 20), followed by incubation in primary antibody diluted 1:1000 in blocking buffer for 1 h at 25 °C. The following antibodies were used: rabbit polyclonal anti-Zic2 (Abcam ab10550), rabbit polyclonal ant-EphB1 (Abnova PAB3018), rabbit polyclonal anti-EphA7 (Abcam ab5400) and rabbit polyclonal Anti-GST IgG (Millipore, #06-332). Membranes were washed in blocking buffer, then incubated with HRP-conjugated secondary antibody (Anti-rabbit IgG, HRP-linked Antibody, Cell Signalling, Beverly, MA, USA, or Anti-Goat IgG-Peroxidase, Sigma-Aldrich) diluted 1:10,000 in blocking buffer for 2 h at 25 °C, followed by three washes inTris buffer. Chemiluminescence was elicited by incubation of the membrane in Luminata Crescendo Western HRP Substrate (Millipore), and detected using a digital luminescence detection system (Alpha Innotech Fluorochem SP Imaging System).

After antibody detection of candidate interacting proteins, membranes were stripped (62.5 mM Tris–HCl, 2% SDS, 100 mM β-mercaptoethanol) and re-probed with anti-GST antibody to determine whether all samples were incubated with the same amount of GST fusion protein, and to ensure that the Ten-m3-GST construct had not been degraded during the pull-down assay. Band sizes were determined by comparison to standard markers.

## Results

### Ten-m3 deletion affects the initial ingrowth of ipsilateral retinogeniculate axons

We have previously shown that deletion of Ten-m3 dramatically affects the distribution of ipsilateral RGC axon terminals in both the dLGN and SC in mature animals [14, 23], but its impact on the formation of the projection has not been reported. Knowledge regarding if and how the distribution of ipsilateral axons is perturbed during their initial ingrowth to target structures may provide important clues regarding the roles and potential interactions of Ten-m3 with other guidance cues.

The SC has been the primary model for investigation of visual mapping mechanisms. We, however, chose to focus on ingrowth to the dLGN for a number of reasons: in the dLGN, ipsilateral RGC projections normally destined for the dorsomedial (Ten-m3 rich) dLGN must grow past the ventrolateral (Ten-m3 poor) region. This contrasts with the SC where the RGC axons grow in from the rostral border, perpendicular to the high-medial to low lateral Ten-m3 gradient [23]. Further, in the SC axons typically grow substantially past the area they will eventually target [5, 23], which could make pathfinding deficits during the initial ingrowth harder to detect. In addition, the ipsilateral projection to the dLGN is both larger and more highly stereotyped in WT mice and shows a more robust phenotype in mature Ten-m3 KOs than the projection to the SC [14, 23]. Together, these factors suggested that the dLGN would provide a more informative structure upon which to base our anatomical studies in Ten-m3 KOs. We therefore examined the development of the retinogeniculate projection using two-colour anterograde tracing of ipsilateral and contralateral axons between P0 and P10.

**Fig. 1:**
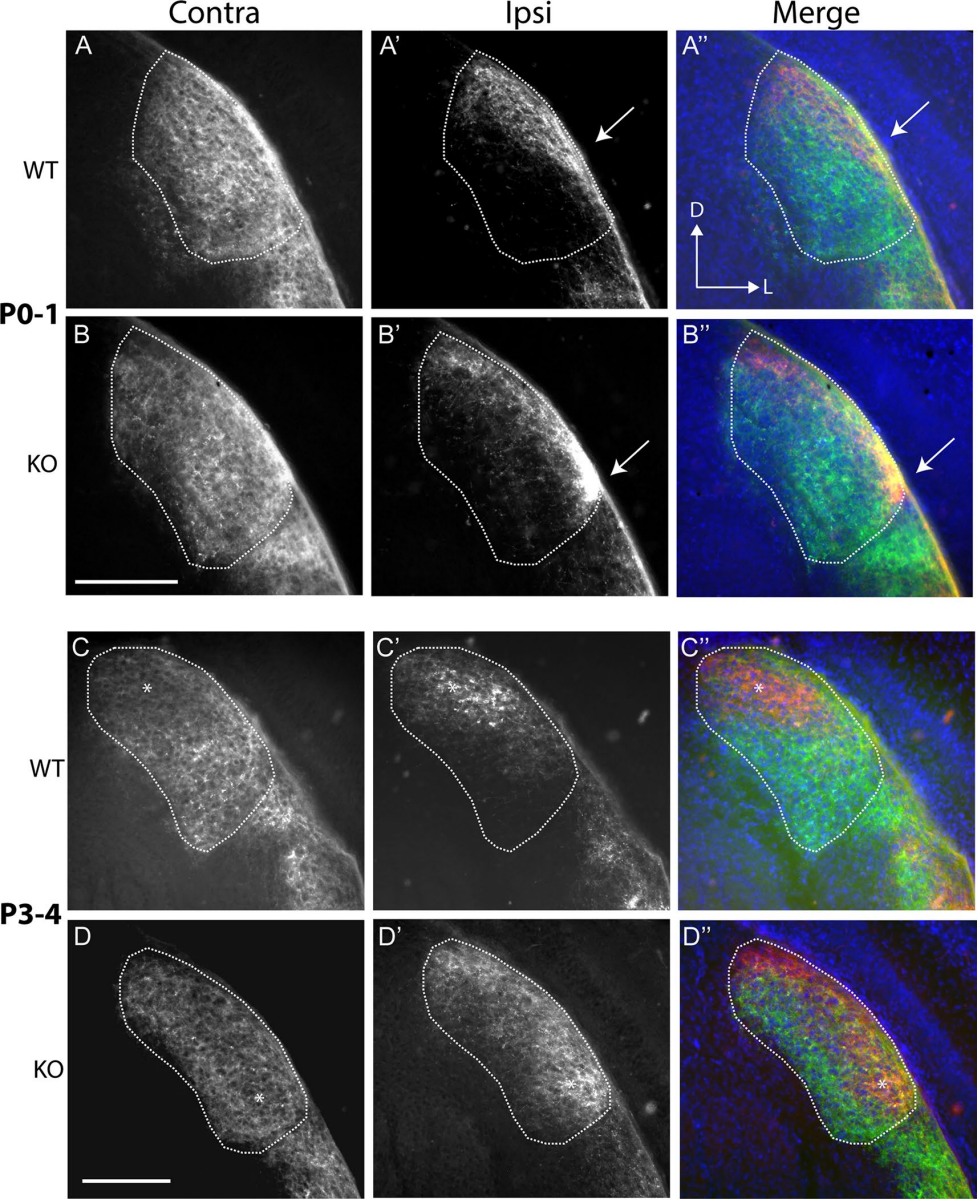
Ipsilateral retinal axons penetrate ventral dLGN from their early ingrowth. Coronal sections through the dLGN following the transport of fluorescent CTB from the eye. Ages and genotypes as marked. Left hand column shows contralateral (Contra) projections, while the middle column reveals ipsilateral (Ipsi) terminals. A merged image is shown on the right (green: contra; red: ipsi; blue DAPI counterstain). **A**, **B** In P0–1 WTs (**A**) contralateral axons had invaded and filled the body of the dLGN. Ipsilateral axons had a much more restricted distribution within the nucleus (**A**′). Axons tended to stay confined to the optic tract overlying the ventral part of the dLGN. Upon entering (arrow), they then fanned out slightly but targeted the dorsomedial segment of the nucleus. The relationships between the two groups of axons can be seen more clearly in the merged image (**A**″). In KO mice (**B**), contralateral axons similarly filled the dLGN. Ipsilateral axons (**B**′), however, showed a very different pattern to WTs. Most notably, the axons entered the dLGN near its ventrolateral border (arrow). The projections extended along the DM-VL extent of the nucleus, as seen in the merged image (**B**″). **C**, **D** By P3–4, WT (**C**) contralateral label was reduced in the dorsomedial segment compared to other regions of the nucleus (*) and the ipsilateral label had begun to consolidate in this area (**C**′, **C**″). In Ten-m3 KO mice (**D**), there was a reduction in the density of contralateral label in a band which ran the length of the nucleus, including the ventral area (*). Ipsilateral label was beginning to consolidate in this region (**D**′, **D**″). Dorsal (*D*) is to the top and lateral (*L*) to the right of each image, as marked in **A**″. Scale bars: 200 μm

Anterograde tracing in P0–1 WTs revealed that contralateral axon terminals filled the bulk of the dLGN (Fig. 1A), whereas ipsilateral axons had a much more restricted distribution (Fig. 1A′, A″). Ipsilateral axons tended to remain confined to the optic tract over the ventral part of the dLGN, and innervated the nucleus only when they approached its dorsal half (Fig. 1A′, A″; arrow). Once they entered the dLGN, they fanned out slightly, but consistently targeted the dorsomedial region. Thus, WT ipsilateral axons displayed evidence that they avoided the ventral half of the nucleus from this early developmental stage. In KO mice, while contralateral axons filled the nucleus as in WTs (Fig. 1B), the distribution of ipsilateral axons was markedly different (Fig. 1B′, B″). Notably, they did not remain restricted to the optic tract in the ventral portion of the dLGN: within the nucleus, ipsilateral axons in KOs were considerably more widely distributed along the DM-VL axis compared to WTs, with a particularly high concentration of signal often visible near the ventral pole (Fig. 1B′, B″, arrow).

By P3–4 the first hints of segregation were beginning to appear with a slightly reduced intensity of labelling discernible in the dorsomedial region of the contralateral projection in WTs (Fig. 1C, *) corresponding to the location of the ipsilateral terminals (Fig. 1C′, C″, *). The ipsilateral projection remained restricted to the dorsal segment of the nucleus (Fig. 1C′, C″). In Ten-m3 KOs (Fig. 1D, D″), ipsilateral axons were again distributed along the entire DM-VL axis of the nucleus. Contralateral axons filled the bulk of the dLGN but showed a reduced labelling intensity corresponding to the position of the ipsilateral axons even in the ventrolateral part of the nucleus (Fig. 1D, *) suggesting that despite the profound mismapping of the ipsilateral projection, the process of segregation was conserved.

**Fig. 2:**
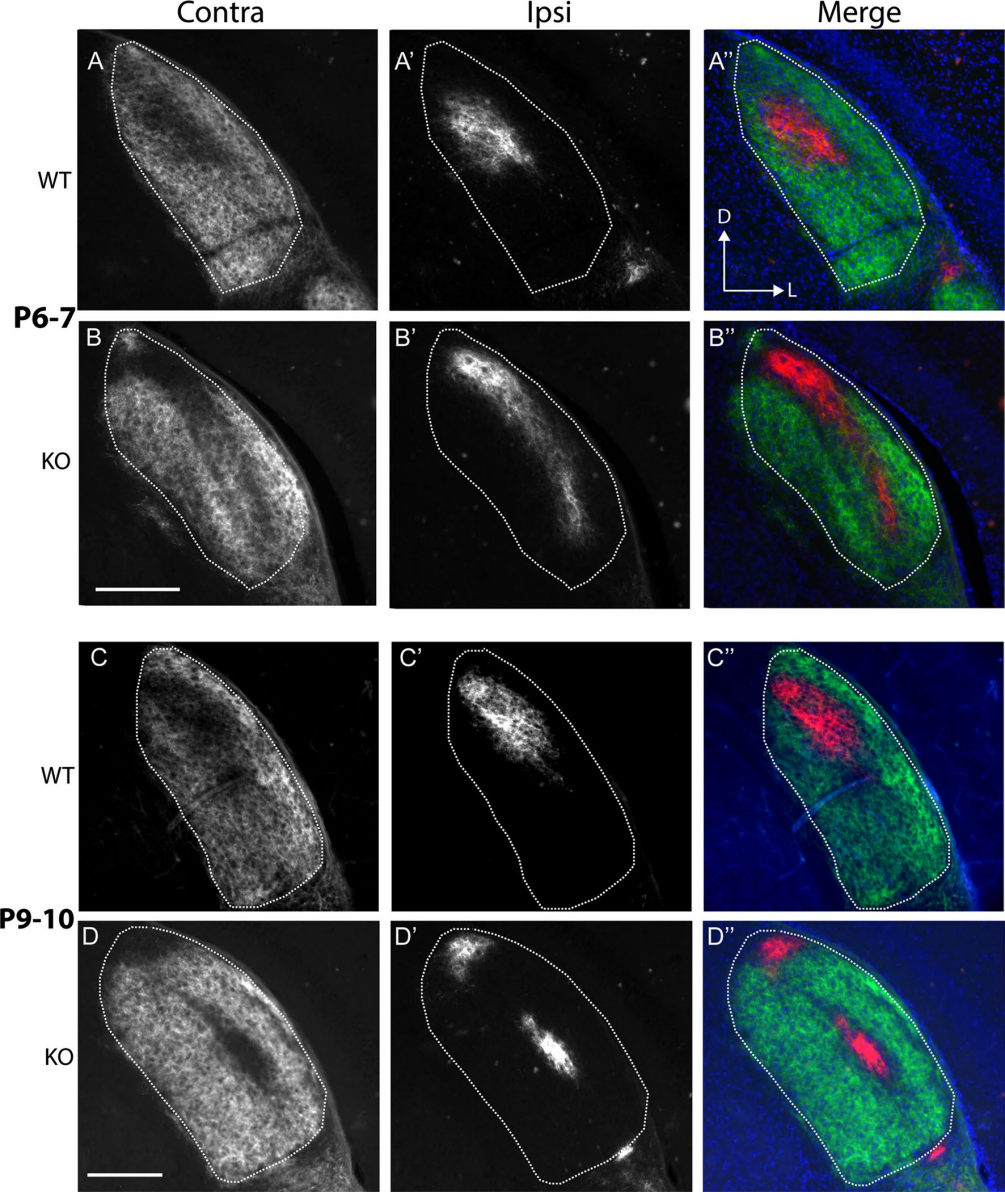
Segregation of ipsilateral and contralateral retinal axons proceeds in Ten-m3 KOs despite ipsilateral mismapping. Conventions are the same as for Fig. 1 but for P6–7 and P9–10 mice as marked. **A**, **B** In P6–7 WTs (**A**), contralateral label showed a clear reduction in a patch within the dorsomedial part of the dLGN. Ipsilateral label was confined to this region (**A**′, **A**″). In Ten-m3 KOs (**B**) the ipsilateral label formed a condensed band that stretched along much of the DM-VL extent of the nucleus (**B**′, **B**″). The contralateral label showed a complementary pattern (**B**). **C**, **D** By P9–10, projections look largely adult-like in WT mice, with a condensed ipsilateral zone situated dorsomedially (**C**′, **C**″), and contralateral terminations filling the rest of the nucleus (**C**). In Ten-m3 KO mice (**D**) the mature pattern was also apparent. The ipsilateral projection persists as a condensed band of terminals which extends into the ventrolateral dLGN (**D**′, **D**″) and contralateral terminals avoiding this region (**D**). Dorsal (*D*) is to the top and lateral (*L*) to the right of each image, as marked in **A**″. Scale bars: 200 μm

By P6–7 in WTs (Fig. 2A, A″), the ipsilateral projection appeared more condensed and occupied a distinct patch in dorsomedial dLGN (Fig. 2A′, A″), with contralateral terminals largely absent from this region (Fig. 2A). In Ten-m3 KOs (Fig. 2B, B″), ipsilateral axons had also become more clustered to form a clear terminal zone (Fig. 2B′, B″). The shape of this region was distinct from WTs, however, in that it was much narrower and more elongated along the DM-VL axis. Contralateral axons (Fig. 2B) largely avoided this area, including the more ventral part which does not normally receive ipsilateral innervation.

By P9–10 (Fig. 2C, C″), the organisation of binocular terminals in WTs resembled that seen in mature animals, with a dense ipsilateral patch in dorsomedial dLGN and minimal overlap of ipsilateral and contralateral projections. The projection in Ten-m3 KOs (Fig. 2D, D″) also resembled that seen in adults in terms of clustering of the ipsilateral terminals and their segregation from contralateral projections. Notably, the ectopic location of ipsilateral axons in Ten-m3 KOs persisted, along with the corresponding avoidance of this region by contralateral terminals. In some sections the ipsilateral label appeared to form two distinct patches, but inspection of nearby sections revealed that the patches were a continuous strip of label.

**Fig. 3:**
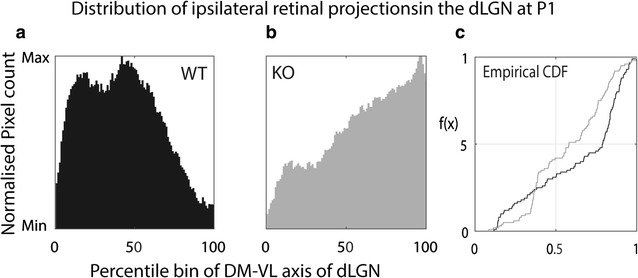
Quantitative analysis confirms altered distribution of ipsilateral terminals in Ten-m3 KOs during ingrowth to the dLGN. **a** Summed distribution of ipsilateral terminals along the dorsomedial-ventrolateral axis of the dLGN, normalized to peak values in WTs at P0. Distribution of labelling along this axis is divided into percentile bins. In WT mice, the bulk of the label is seen in the dorsal half of the nucleus, peaking at around 40% of this axis. **b** As for **a**, but for Ten-m3 KOs. The ipsilateral retinogeniculate terminals show a markedly different distribution which peaks at around 95% of the dorsomedial-ventrolateral axis of the dLGN. Markedly less labelling is present in the dorsal half of the nucleus. **c** Empirical cumulative distribution function (CDF) shows a significant shift for Ten-m3 KOs (grey line) compared to WTs (black line). These distributions were significantly different (Kolmogorov–Smirnov test; D = 0.029; p < 0.001)

In order to quantitate these pathfinding differences we focused on material from P0–1 animals, as this time-point is most closely reflective of changes in axonal guidance. Analysis of the distribution of ipsilateral terminals along the DM-VL axis of the dLGN at P1 confirmed a clear difference between genotypes, with a marked increase in the density of ipsilateral label in the ventral part of the nucleus in KOs compared to WTs (Fig. 3). These distributions were significantly different (Kolmogorov–Smirnov test; D = 0.29; p < 0.001). These data indicate that the distribution of ipsilateral RGC axons in Ten-m3 KOs differs substantially from that of WTs from the time of their ingrowth into the dLGN. Notably, ipsilateral axons invaded the ventrolateral part of the nucleus in Ten-m3 KOs whereas they avoided this region in WTs.

### Identification of downstream signalling pathways

The atypical ingrowth of ipsilateral axons into the ventrolateral segment of the dLGN in KOs cannot be readily explained by the removal of a homophilic attractant that is normally expressed at high levels in the dorsal part of the nucleus. Rather, this atypical patterning seems more consistent with what might be expected following the removal of a repellent molecule from ventral dLGN. Previous studies have suggested that the graded expression of ephrinA2/5 along the DM-VL axis prevents EphA5, 6 expressing ipsilateral axons from ventrotemporal retina invading the ventral part of the nucleus [9]. We therefore assessed whether EphA or ephrinA expression is altered in retinal targets in Ten-m3 KOs.

**Fig. 4:**
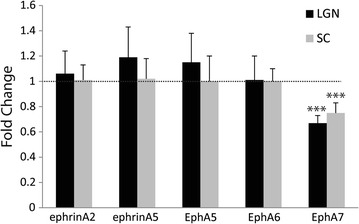
*EphA7* expression is down-regulated in dLGN and SC of Ten-m3 KOs. Realtime PCR revealed significant down-regulation of *EphA7* mRNA in the dLGN (0.67 fold, p < 0.001), and SC (0.75 fold, p < 0.001) of Ten-m3 KOs compared to WTs. No changes in gene expression were detected in any other EphA/ephrinA tested. Graph shows relative fold change ± 1SE, normalised to *Gapdh* and in comparison to WT controls. ***Denotes p < 0.001

To identify candidates for more detailed analyses, we examined whether the EphA receptors and ephrinA ligands which have been previously shown to regulate topographic mapping, exhibited changes in expression using qPCR on samples from both the dLGN and SC (this structure was included as topographic mapping gradients have been most extensively described here) of P0 WT and Ten-m3 KO mice. We found a similar and significant decrease in the relative expression levels of *EphA7* receptor mRNA in both SC (fold change 0.75 ± 0.08; p < 0.001, Pairwise fixed random reallocation test; Fig. 4) and dLGN (fold change 0.67 ± 0.06; p < 0.001, Pairwise fixed random reallocation test; Fig. 4) of samples from Ten-3 KOs compared to WTs. None of the other EphA (*EphA5* and *EphA6*) or ephrinA (*ephrin A2* and *ephrinA5*) transcripts tested showed detectable differences in expression between genotypes (Fig. 4).

### EphA7, but not ephrinA5, expression gradients are markedly reduced throughout the central visual pathway of Ten-m3 KOs

**Fig. 5:**
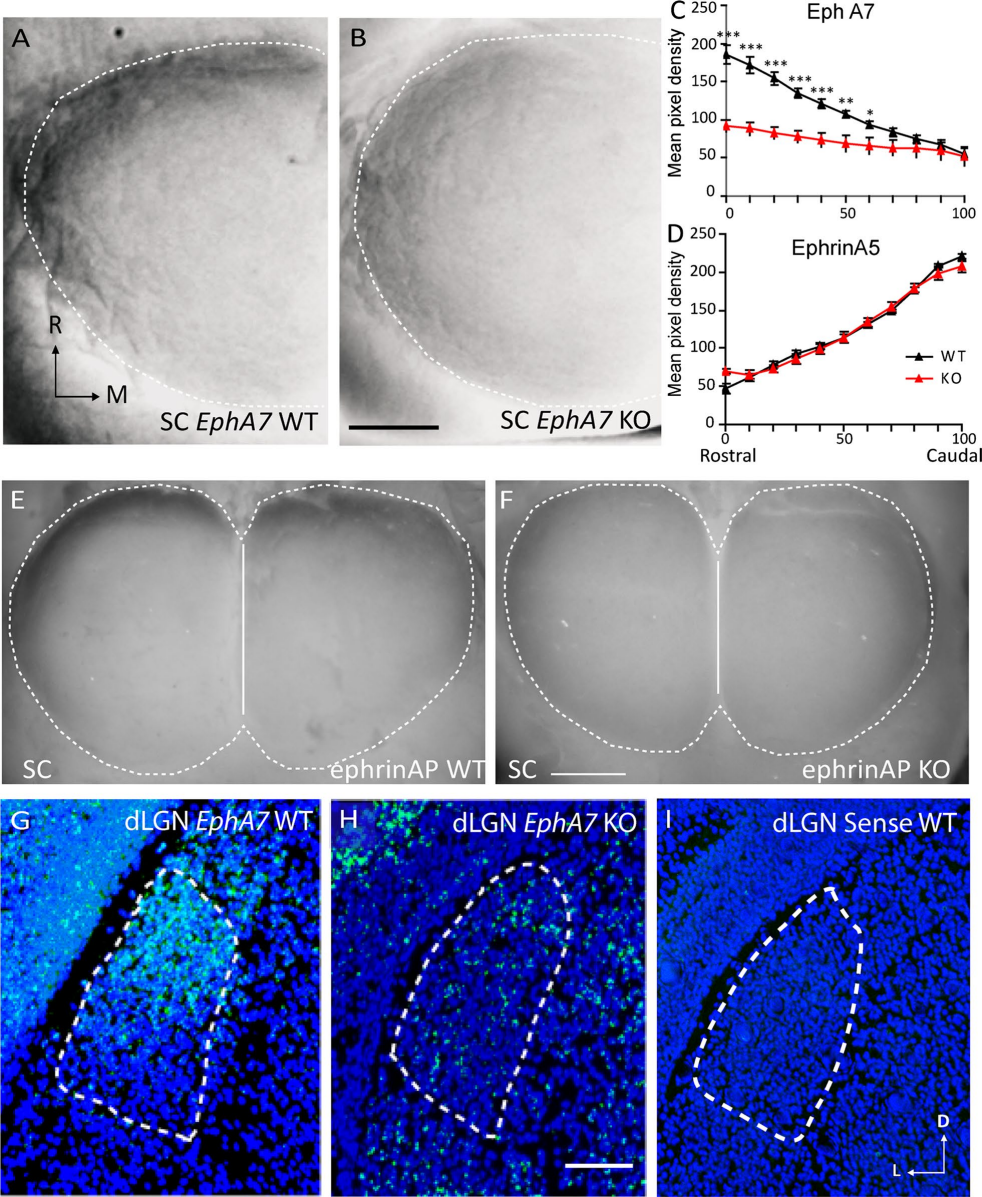
*EphA7* expression gradients are reduced in SC and dLGN of Ten-m3 KOs. **A**–**D** In situ hybridisation for *EphA7* mRNA on whole mounts of SC from WT (**A**) and Ten-m3 KO (**B**) P0 mice. One lobe of the SC is shown. *M* medial; *R* rostral. In WTs high expression is apparent in the rostral SC (top of image) and decreases caudally. In Ten-m3 KOs *EphA7* expression is much lower rostrally than in WT and a notably shallower gradient is present across the rostrocaudal axis. The dotted line denotes the borders of the SC. The midline is on the right side of the images. **C** Semi-quantitative analysis of all in situ hybridisation whole mount cases examined (n = 5–6 SCs each from three KOs and 3WTs). WT data show a linear high rostral to low caudal gradient (black trace). KO data (red trace) exhibit a much shallower gradient of expression with significantly lower values compared to WT across the most rostral 60% of the nucleus (univariate ANOVA, p < 0.001, interaction F(10, 99) = 6.789, p < 0.001; pairwise comparisons across each region of equal length show significant differences across the most rostral 60% of the nucleus). **D** Similar measurements obtained following in situ hybridisation of whole mounts SCs with an *ephrinA5* probe. Whole mounts of SC reveal identical high-caudal to low rostral gradients of expression in Ten-m3 KOs as seen in WTs. ***p < 0.001; **p < 0.01; *p < 0.05. Scale bar: 250 μm, applies to **A**, **B**. **E**, **F** Whole mounts of SC stained using an ephrinA alkaline phosphatase affinity probe to reveal the binding activity of EphA receptors. In WTs (**E**), staining is darker rostrally (top of image) and diminishes more caudally. In KOs (**F**) expression is lower at the rostral end and largely uniform along the rostrocaudal axis. Dotted white line denotes borders of SC, solid line marks midline. Scale bar: 500 μm; applies to **E**, **F**. **G**–**I** Coronal sections through the dLGN at P0 following an in situ hybridisation reaction for *EphA7* mRNA. In the WT (**G**) expression is high dorsally and reduced ventrally. Expression levels are dramatically reduced in KO (**H**) with very low levels of staining and no apparent gradient. The WT sense control (**I**) also shows low levels of staining. Dotted white line marks borders of dLGN. Dorsal is to the top and medial to the right of images. Scale bar: 100 μm; applies to **G**–**I**

The EphA7 receptor is reported to be expressed in a high rostral to low caudal gradient in the SC where it participates in retinotopic mapping [8]. In neonatal WTs in situ hybridisation revealed the expected expression pattern of *EphA7* mRNA (Fig. 5A). In KOs, however, expression was consistently diminished (Fig. 5B), and showed little evidence of a rostrocaudal gradient. Semi-quantitative analysis confirmed this observation (Fig. 5C). Expression levels were significantly reduced in KOs over the most rostral 60% of the SC [univariate ANOVA, genotype (WT vs. KO) * region (anterior to posterior axis of the SC divided into equal segments) interaction F(10, 99) = 6.789, p < 0.001, η^2^ = 0.407; pairwise comparisons between genotype at each location: WT vs. KO, 0–4th region: p < 0.001; 5th region: p = 0.002; 6th region: p = 0.019; 7th to 10th region: p > 0.05; Fig. 5C; n = 5–6 SCs each from 3KOs and 3WTs), but converged towards the caudal pole. In contrast to the diminished *EphA7* gradient in the SC of Ten-m3 KOs, we found that the low-rostral to high caudal *ephrinA5* gradient was maintained in the SC, identical to WTs (Fig. 5D).

The binding of alkaline-phosphatase (AP) tagged ephrinA receptor probes (ephrinA-AP) reflects the distribution and binding activity of EphA receptor protein [34]. The high rostral to low caudal distribution present in WTs (Fig. 5E) was absent in Ten-m3 KOs (Fig. 5F). Since EphA7 is thought to be the most significant contributor to this gradient [8], these results suggest the changes to *EphA7* mRNA levels lead to changes in protein expression.

We also used in situ hybridisation to examine the pattern of *EphA7* expression in the dLGN of Ten-m3 KOs at P0. The previously reported [9] prominent high-dorsal to low-ventral gradient present in the dLGN of WTs (Fig. 5G) was absent in Ten-m3 KOs (Fig. 5H). No signal was present in sense controls (Fig. 5I).

### EphA7 is expressed in retinal ganglion cells

**Fig. 6:**
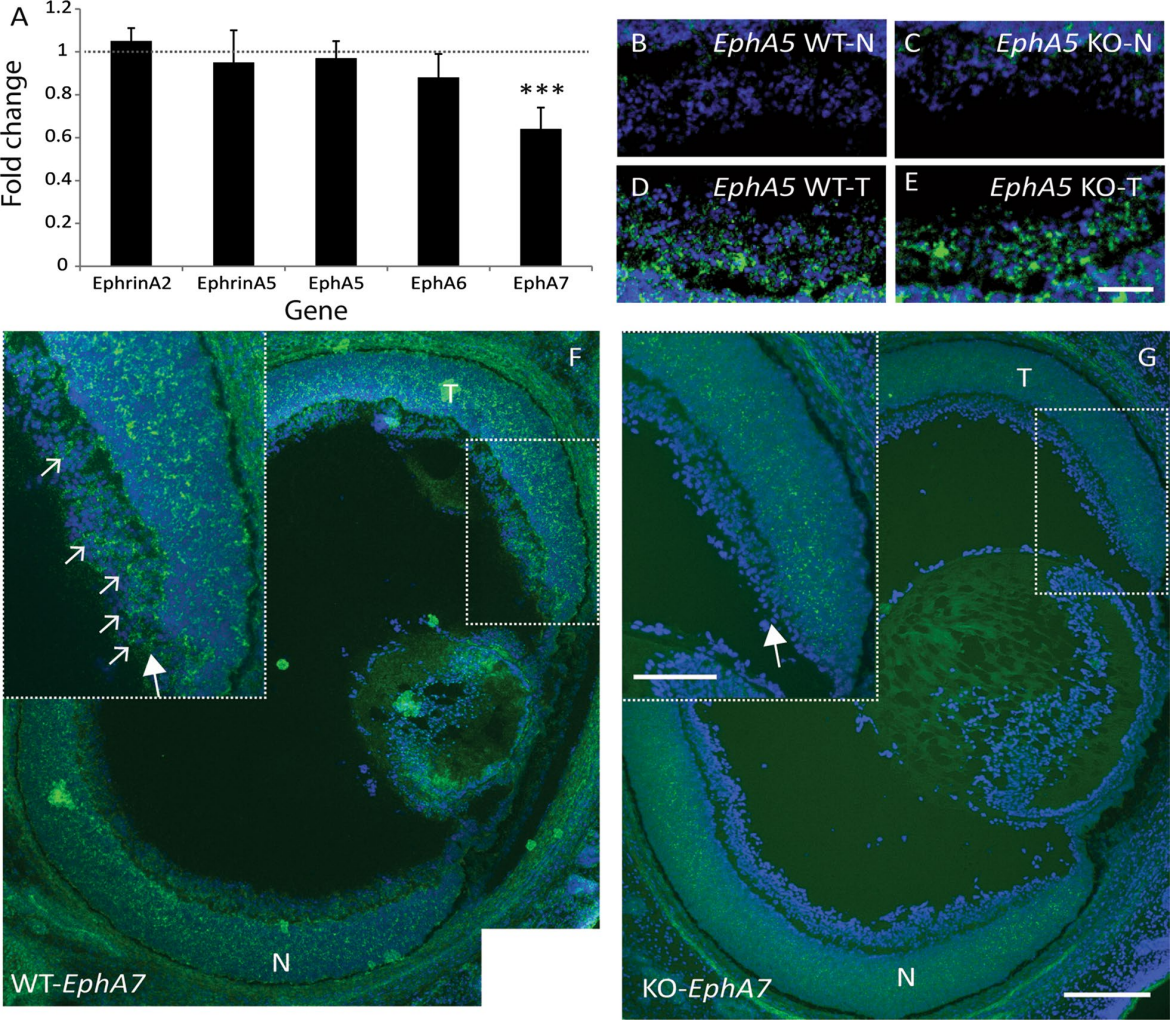
EphA7 is usually expressed in the ventrotemporal retinal crescent and is reduced in Ten-m3 KOs. **A** Realtime qPCR revealed significant down-regulation of *EphA7* mRNA in the retina (fold change 0.64 ± 0.01, p < 0.001) of Ten-m3 KOs compared to WTs. No change in expression was detected in any of the other members of the EphA/ephrinA family tested. Graph shows relative fold change ± 1SE, normalised to *Gapdh* and in comparison to WT controls. Statistical significance is denoted by (*): p < 0.05*, p < 0.01**, p < 0.001***. **B**–**E** In situ hybridisation for *EphA5* (green staining) on retinal sections for WT (**B**, **D**) and Ten-m3 KO (**C**, **E**) revealed no differences in expression pattern in the RGC layer. Signal was low in samples from nasal (*N*) retina from both WT and KO (**B**, **C**). Similarly high levels of expression observed in samples from temporal (*T*) retina of both genotypes (**D**, **E**). In situ hybridisation signal is superimposed on DAPI stain (blue) to reveal cell nuclei. Scale bar: 50 μm; applies to **B**–**E**. **F**, **G** In situ hybridisation on horizontal retinal sections for *EphA7*. In sections from WT (**F**), *EphA7* (green staining) is expressed in a subset of cells in the RGC layer from far temporal (*T*) retina [dotted square; inset (large arrow)]. A fluorescent Nissl counterstain (DAPI) is shown in blue. Small arrows highlight a subset of *EphA7* positive cells in this region. In an equivalent section from a Ten-m3 KO retina (**G**), *EphA7* expression is markedly reduced, including in the RGC layer (arrow in inset). No *EphA7* positive cells are visible in this region. Scale bar: 200 μm; scale in inset: 100 μm; applies to **F** and **G**. *N* nasal

Our data identified EphA7 as a potential downstream effector of Ten-m3 signalling in central visual structures. Since EphA–ephrinA interactions are largely repellent in the retinofugal system [3, 4, 6, 7, 35], the reduced *EphA7* expression in rostral SC and dorsal dLGN does not easily explain the mismapping of ipsilateral retinal axons present in Ten-m3 KOs. Therefore, we also examined the *EphA*–*ephrinA* mRNA expression profile in the retina. While none of the other *EphA* receptors *or ephrinA* ligands tested were significantly altered (Fig. 6A), *EphA7* mRNA was significantly down-regulated in Ten-m3 KOs (fold change 0.64 ± 0.10, p = 0.001; Pairwise fixed random reallocation test). This was surprising as previous studies have indicated that EphA7 is either not expressed in retina [8], or is absent from RGCs [36]. To assess the spatial expression profile of retinal *EphA7*, we performed in situ hybridisation on horizontal sections through the eye of P0 WT and Ten-m3 KO. *EphA5* expression was also investigated as a positive control.

*EphA5* mRNA exhibited a high-temporal to low-nasal distribution, including in the RGC layer, of WT mice (Fig. 6B, D), consistent with published studies [3]. The expression pattern of *EphA5* in KOs (Fig. 6C, E) was indistinguishable from WT.

*EphA7* was also found to be expressed in the RGC layer of P0 WT (Fig. 6F). Interestingly, its distribution was more restricted than that of *EphA5* and could only be detected in the temporal region of ventral retina (Fig. 6F and inset; arrows). *EphA7* expression in the RGC layer in Ten-m3 KOs was notably lower than in WTs (Fig. 6G and inset), consistent with the qPCR data. Together, our findings suggest that *EphA7* is present in a region which corresponds to the origin of the ipsilateral retinal projection in WTs, and is down-regulated in Ten-m3 KOs.

### The intracellular domain of Ten-m3 binds with Zic2 and regulates the Zic2–EphB1 pathway

It is possible that Ten-m3 may also interact with other genes and signalling pathways to regulate ipsilateral retinal mapping. Previous studies have demonstrated interactions between Zic1 and Ten-m2 [25]. The Zic2–EphB1 molecular pathway is of particular interest here as it has been shown to be critical for the specification and guidance of ipsilateral retinal axons [1, 37]. We therefore investigated possible Ten-m3 binding interactions with Zic2 and/or EphB1. Since a recent study has found differential expression of Zic1 and Zic3 in ipsilaterally versus contralaterally-projecting RGCs [29], we also investigated whether either of these molecules had the ability to bind to the intracellular domain of Ten-m3.

**Fig. 7:**
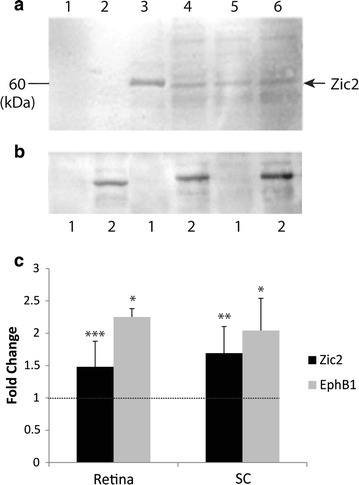
Ten-m3 interacts with the Zic2–EphB1 signalling pathway. **a** Western blot showing immunodetection of a 60 kDa band corresponding to the expected size of Zic2 following GST pull-down assay with mouse P1 whole brain lysates; sepharose 4B beads (*lane 1*), GST alone (*lane 2*), Ten-m3-GST fusion protein probe (*lane 3*), and supernatants of beads only (*lane 4*), GST alone(*lane 5*), and Ten-m3-GST (*lane 6*). The 60 kDa Zic2 band can be observed in the lane containing Ten-m3-GST (*lane 3*) but not beads (*lane 1*) or GST alone (*lane 2*). Additionally, immunoreactivity for Zic2 can be seen in all supernatants (*lanes 4–6*) confirming that Zic2 was present and suggesting the Ten-m3-GST probe concentration was unable to bind all Zic2 present in the lysate. **b** As for **a** following repeat pull-downs using lysate from three individual animals. *Lane 1* GST alone; *Lane 2* Ten-m3-GST fusion protein probe. These findings confirm a reliable and specific interaction between Zic2 and the intracellular domain of Ten-m3. **c** Realtime qPCR revealed significant up-regulation of *Zic2* in retina (1.5 fold, p < 0.001), and SC (1.7 fold, p = 0.001) in Ten-m3 KOs compared to WTs. These results suggest that Ten-m3 may modulate the expression of Zic2 by repressing transcription. A correlated increase in *EphB1* mRNA was also observed both in the retina (fold change 2.25 ± 0.13; p = 0.016) and SC (fold change 2.04 ± 0.5; p = 0.011). Graph shows relative fold change ± 1SE, normalised to *Gapdh* and in comparison to WT controls. Statistical significance is denoted by (*): p < 0.05*, p < 0.01**, p < 0.001***

We first performed a Glutathione-S-Transferase (GST) pull-down assay using the intracellular domain of Ten-m3 and whole brain lysate from P1 mice followed by Western blotting for the proteins of interest. Upon probing for Zic2 we consistently found a 60 kDa band which corresponds to the known molecular weight of this protein in the lane containing the GST-Ten-m3 construct (lane 3; Fig. 7a). No band corresponding to Zic2 was found in the control lanes containing sepharose beads or the GST construct alone (lanes 1 and 2; Fig. 7a). Zic2 immunoreactivity was present in the supernatant under all conditions (lanes 4–6; Fig. 7a) confirming that it was present in all samples. Repeat pull-downs were performed using lysate from five independent samples (Fig. 7b; 3 examples shown here) and produced identical results, confirming the interaction. In contrast, bands corresponding to the expected sizes of Zic1, Zic3, EphA7 and EphB1 could not be detected in GST-Ten-m3 construct pull-down lanes, although these bands were detected in the whole brain lysate and supernatant in each case, indicating that these proteins were also present in the samples (Additional file 1: Fig. S1).

Real-time quantitative PCR showed that there was a significant up-regulation of transcription factor *Zic2* in samples taken from P1 retina (fold change 1.48 ± 0.397; p < 0.001) and SC (fold change 1.69 ± 0.413, p = 0.001; Fig. 7c) of Ten-m3 KOs compared to WTs. A significant level of up-regulation of *EphB1* was also observed in both the retina (fold change 2.25 ± 0.13; p = 0.016) and SC (fold change 2.04 ± 0.5; p = 0.011; Fig. 7c) of KOs compared to WTs.

**Fig. 8:**
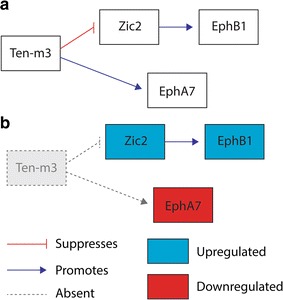
Schematic diagram illustrating potential interactions and changes in Ten-m3 KOs. **a** In WT mice, Ten-m3 acts to repress *Zic2*, a promoter of *EphB1* transcription. *Ten*-*m3* also promotes *EphA7* expression. These interactions may be direct or indirect. Other interactions are also possible. For example, Ten-m3’s regulation of *EphA7* may involve or be downstream of its interaction with Zic2 (not shown). **b** In Ten-m3 KO mice the repression of *Zic2* is removed and thus Zic2 is upregulated which in turn drives increased expression of *EphB1*. The positive regulation of *EphA7* by Ten-m3 is also removed causing a reduced expression of *EphA7* compared to WT

These data thus demonstrate that the Zinc finger transcription factor Zic2 can bind to the intracellular domain of Ten-m3. Further, the correlated increase levels of Zic2 and Eph1 in Ten-m3 KOs suggest that this interaction may usually lead to the inhibition of this molecular signalling pathway (see Fig. 8).

## Discussion

This study has demonstrated that ipsilateral retinal axons exhibit a markedly altered trajectory during their initial growth into the dLGN of Ten-m3 KOs, invading the ventrolateral region of the nucleus from which they are usually repelled by high levels of ephrinA. This occurs despite the fact that expression of mRNA for *EphA*–*ephrinA* signalling molecules is largely intact in Ten-m3 KOs. A notable exception was the *EphA7* receptor which was specifically and significantly down-regulated in the visual pathway of these mice. Our data shows that *EphA7* is normally expressed in a subset of cells in the RGC layer of ventrotemporal retina, and this expression is markedly reduced in Ten-m3 KOs. Finally, using a protein pull-down assay we identified Zic2 as a binding partner for the intracellular domain of Ten-m3 and demonstrated that *Zic2* and *EphB1* are both significantly up-regulated in Ten-m3 KOs. Together, these results suggest that Ten-m3 may help to regulate the ingrowth of ipsilateral retinal axons to the dLGN by promoting *EphA7* transcription which contributes to the repulsion of these axons from the ephrinA-rich ventrolateral portion of the nucleus. The interaction demonstrated with Zic2, whose expression specifies ipsilateral fate in the retina [1] provides a mechanism by which Ten-m3 may exert its specific but profound effect on the targeting of uncrossed retinal axons. Figure 8 schematically illustrates the interactions of Ten-m3 suggested by our data.

### Aberrant initial ingrowth of ipsilateral retinal axons to the dLGN in Ten-m3 KOs

Our WT neuroanatomical data indicate that contralaterally-projecting retinal axons initially fill the dLGN by P1, and then gradually retract from the ipsilateral recipient zone in the dorsomedial part of the nucleus over the subsequent 1–2 weeks. Ipsilateral axons tended to avoid the ventrolateral region of the dLGN in WTs, remaining confined to the optic tract overlying this area, and targeted the dorsomedial part of the nucleus from their first ingrowth. This is in agreement with previous studies [9, 38]. In Ten-m3 KOs, contralateral axons followed the same developmental pattern as seen in WTs. Ipsilateral axons, however, showed a markedly different pattern, exiting the optic tract to invade the ventrolateral dLGN from the outset. Despite the altered targeting of these projections, the time-course and manner of segregation from their contralateral counterparts appeared identical to WTs. This indicates that the targeting of ipsilateral retinal projections and their segregation from contralateral inputs can proceed independently, even in ectopic locations. The adult pattern of ipsilateral mismapping in Ten-m3 KOs [14] correlates well with, and probably arises as a direct consequence of these early guidance deficits.

### The role of Ten-m3 in the formation of binocular circuits is likely to involve non-homophilic interactions

Recent studies have emphasised the importance of direct homophilic interactions of Ten-m proteins in synaptic matching [16, 17, 21]. Although the topographically corresponding gradients of Ten-m3 in visual structures are consistent with this [14, 23], the axonal guidance defects we observed here cannot be readily explained by synaptic level interactions alone. In particular, the early exit of the ipsilateral RGC axons from the optic tract in the ventrolateral dLGN, a region from which they are usually excluded via EphA–ephrinA mediated repulsion [9], cannot be easily accounted for by the removal of a synaptic matching molecule in the dorsal dLGN. It therefore seems likely that Ten-m3 acts through other mechanisms to regulate the targeting of ipsilateral retinal axons. Higher-resolution studies will be required to assess whether Ten-m3, possibly in conjunction with other members of the Ten-m family, plays additional roles in synapse formation in mammals.

### EphA7 is a downstream target of Ten-m3

Previous studies have shown that appropriate targeting of ipsilateral retinal projections is dependent on the presence of ephrinA molecules, which are usually highly expressed in ventrolateral dLGN [9]. Thus, the altered ingrowth pattern we observed here in Ten-m3 KOs is suggestive of alterations in EphA–ephrinA signalling. Indeed, *EphA7* mRNA expression was consistently reduced in the retina, SC and dLGN in Ten-m3 KOs at around the time of birth. Although we did not assess changes in gene expression at later stages, given that both Ten-m3 [14, 22, 23] and *unpublished observations*, CAL) and EphA7 [8] maintain their expression patterns throughout the first postnatal week it is likely that the differences we observed would be maintained throughout this time. The consistency of the effect across different areas of the visual pathway suggests that Ten-m3 is an important regulator of EphA7 expression. Protein pull-down experiments demonstrated that the intracellular domain of Ten-m3 and EphA7 do not undergo protein–protein interactions, but this does not exclude the possibility that Ten-m3 protein may indirectly regulate transcription of EphA7 mRNA. Given that the expression patterns of these genes exhibit orthogonal gradients (in the SC, for example, Ten-m3 has a high-medial to low lateral gradient compared to the high rostral to low caudal gradient of EphA7), this is likely to be the case. It should be noted, however, that the maximum overlap of these genes is in the region of the visual pathway associated with ipsilateral projections.

The identification of EphA7 as a downstream target of Ten-m3 (Fig. 8) was to some extent unexpected, particularly in the retina where EphA7 has been variably reported as either present but not expressed in RGCs [36], or completely absent [8]. The discovery that *EphA7* is present in WT retina and reduced in Ten-m3 KOs was shown consistently using both quantitative and qualitative approaches. The difference between our data and that of [8] may be due, at least in part, to the different techniques used. While a previous study employed Western blots to reveal retinal EphA7 expression [8], we instead relied on qPCR and in situ hybridisation. Given that EphA7 is only expressed in a subset of cells from a subregion of retina, Western blots run on whole retina samples may not have had the sensitivity to reveal its presence.

A potential role for EphA7 in the mapping of ipsilateral retinal axons has not been previously demonstrated. EphA7 has been shown to prevent contralateral axons from nasal retina forming ectopic arbors in rostral SC [8]. While contralateral mapping is largely normal in Ten-m3 KOs [23], this has not been explicitly addressed for nasal axons. Interestingly, retrograde tracing suggests that contralateral ventrotemporal retinal axons have enlarged terminal zones in the SC of EphA7 KOs [8]. Further, anterograde tracing reveals that contralateral ventrotemporal axons have rostrocaudally elongated TZs in Ten-m3 KOs [23]. The presence of branching along an increased region of the rostrocaudal SC axis in these animals is consistent with the postulated role of EphA7 in preventing axonal branching rostral to the TZ, suggesting an interplay between these two factors. Future studies should explicitly address the manner in which Ten-m3 and EphA7 interact, as well as EphA7’s role in ipsilateral retinal mapping.

In order for rodents to properly align binocular visual input, ipsilateral and contralateral projections which arise from the same region of retina must map differentially in their target structures (reviewed in [23]). This suggests that ipsilateral and contralateral axons may differentially express topographic guidance molecules. The candidates primarily associated with topographic mapping of the nasotemporal retinal axis, EphA5 and EphA6, are typically described as showing smooth gradients across the retina [3, 7]. It should be noted, however, that since the population of ipsilaterally-projecting RGCs is small and intermixed with the contralateral population [24], potential differences in the expression levels of guidance molecules in this subset of cells could readily have been overlooked. In a recent screen to identify ipsilateral-specific molecules, no members of the EphA family were detected [29], although this may have been due to the early age point assessed in this study.

The profound miswiring of ipsilaterally-projecting RGCs in the presence of normal ephrinA gradients in Ten-m3 KOs, suggests that this population has differential guidance requirements than those previously described for contralaterally-projecting axons. The demonstration of a restricted labelling pattern of EphA7 in a subset of cells in the ventrotemporal region of the RGC layer in WTs raises the intriguing possibility that this axonal guidance molecule is selectively and/or preferentially expressed in ipsilaterally-projecting populations. Indeed, an ipsilateral-specific (or differential) expression of EphA7 would provide a means by which ipsilaterally- and contralaterally projecting RGCs from similar parts of the retina could map appropriately to properly align binocular visual inputs in central targets. Given the specific regulation of EphA7 by Ten-m3, it would also help to explain the profound influence of Ten-m3 on ipsilateral but not contralateral projections [14, 23]. The regulation of EphA7 expression specifically in ipsilaterally-projecting RGCs by Ten-m3 could potentially arise downstream of Ten-m3’s interaction with Zic2. A more detailed expression study using single cell qPCR or transcriptome sequencing would be required to determine the degree to which EphA5, 6, 7, or any other candidate gene is differentially expressed in ipsilaterally versus contralaterally-projecting RGCs.

### Ten-m3 interacts with Zic2–EphB1 signalling pathway

The identification of Zic2 as a binding partner for the intracellular domain of Ten-m3 provides an important mechanistic link between the profound but specific effect of Ten-m3 on ipsilateral projections, and its much broader expression pattern within the retina [14, 23]. The correlated up-regulation of *Zic2* and *EphB1* in Ten-m3 KOs (Fig. 8) is consistent with data showing that Zic2 promotes EphB1 expression to cause repulsion of RGCs at the ephrinB2-rich chiasmatic midline, resulting in the formation of the ipsilateral retinal projection [1, 37].

While more direct evidence is required to confirm the cleavage and translocation to the nucleus of the intracellular domain of Ten-m3, multiple indirect lines of evidence from this and previous studies support this possibility. Firstly, the deletion of Ten-m3 leads to consistent changes in the transcription of multiple axonal guidance molecules at different levels of the visual pathway where Ten-m3 is prominently expressed. Of particular note is the correlated up-regulation of Zic2 and EphB1 (as noted here and consistent with previous work [1, 37]) as well as the down-regulation of EphA7. Secondly, the intracellular domain of Ten-m3 consistently and specifically binds to a nuclear transcription factor which is intimately associated with the specification and guidance of ipsilateral retinal axons [1]. Thirdly, as noted above, the intracellular domain of the highly homologous Ten-m2 has been shown to interact with Zic1 [25]. Finally, the intracellular domain of Ten-m3 contains a potential nuclear localisation signal and cleavage site for its release [27, 28], and this property is shared by all of the other Teneurin family members (Ten-m1 [18, 26], Ten-m2 [39] and Ten-m4 [40]).

Although binding of the intracellular domain of Ten-m3 and Zic2 was reliably detected, the nature of this interaction remains to be determined. In particular it is unclear if the two proteins bind directly with each other, or indirectly as part of a complex. Our finding that Ten-m3 usually acts to suppress Zic2 expression suggests that Ten-m3 may act upstream of Zic2. Further studies will be needed to investigate the exact nature of their relationship.

Zic2 mutants are associated with a reduced ipsilateral projection, and ectopic expression of Zic2 drives an increase in the size of the uncrossed population of RGC axons [1, 37]. At one level it is surprising then that although their terminals are mismapped, the size and spatial origin of the ipsilateral projection in Ten-m3 KO mice is identical to WTs [14]. The cells which form the mature ipsilateral retinal projection are generated between E11 and E16 [41]. One possibility is that the up-regulation of Zic2 in Ten-m3 KOs occurs too late to drive the formation of additional ipsilaterally-projecting axons. This seems somewhat unlikely, however, as Zic2 expression peaks at E16.5 [1] and Ten-m3 is already prominently expressed at this time [23]. Alternately, the up-regulation of Zic2 and EphB1 expression may occur only in cells which usually express Zic2 and EphB1. This interpretation is supported by our results demonstrating that the intracellular domain of Ten-m3 can bind with Zic2. The up-regulation of EphB1 in ipsilaterally-projecting RGCs would be expected to cause them to map more laterally in the SC than is usually the case [42]. We have previously reported this for Ten-m3 KO mice [23], providing further support for this hypothesis.

Interestingly, another closely-related member of the Ten-m family, Ten-m2, is also critical for the formation of the ipsilateral retinal projection via an interaction with EphB1 [43]. Unlike Ten-m3 KOs, however, Ten-m2 KOs display reduced expression of EphB1 with no impact on Zic2. Further, Ten-m2 deletion results in reduced EphB1 expression, and a correlated loss of ipsilaterally-projecting RGCs, but only from the ventral part of the ventrotemporal crescent [43]. Thus, while the current study suggests that Ten-m3 interacts with Zic2 to inhibit Zic2/EphB1 expression, Ten-m2 appears to function either independently or downstream of Zic2 to up-regulate EphB1. Further studies will be required to elucidate the precise relationship between all these molecules.

Our findings, together with previous studies discussed above, point to a molecular network where Ten-m3 and Zic2 interact to play complementary roles in the establishment of binocular visual pathways: Zic2 specifies ipsilateral identity [1] whereas Ten-m3 is required to enable appropriate mapping of these axons such that they align with their contralateral counterparts within their target structures. These processes involve down-stream signalling molecules including, but presumably not limited to, EphA7, Ten-m2 and EphB1.

## Conclusion

This study provides the first evidence of the downstream interactions of Ten-m3. The results help to explain the specificity with respect to laterality and axial direction of action for Ten-m3, two prominent and functionally important features of the Ten-m3−/− phenotype which do not correlate obviously with its expression pattern. The work adds to a growing body of knowledge regarding the molecular interactions which regulate the formation of functional binocular visual circuits.

## Additional file

**Additional file 1: Fig. S1.** Western blots showing immunodetection of proteins corresponding to the expected sizes of Zic1 (A), Zic3 (B), EphA7 (C) and EphB1 (D) following GST pull-down assay with mouse P1 whole brain lysates. In each case the lanes show: sepharose beads (lane 1), GST alone (lane 2), Ten-m3-GST fusion protein probe (lane 3), whole brain lysate positive control (lane 4), and supernatants of beads only (lane 5), GST alone (lane 6), and Ten-m3-GST (lane 7). The prominent bands corresponding to the expected size of each of the proteins can be observed in the whole brain lysate and supernatants of all conditions, but not in any of the pull-down lanes. Most notably, no band is present in the Ten-m3-GST pull-down lane (lane 3), suggesting that there is no direct binding interaction between the intracellular domain of Ten-m3 and any of these molecules.

## Abbreviations

CTB: cholera-toxin subunit B
dLGN: dorsal lateral geniculate nucleus
DM-VL: dorsomedial to ventrolateral
DAPI: 4′,6-diamidine-2′-phenylindole dihydrochloride
GAPDH: glyceraldehyde-3-phosphate dehydrogenase
GST: glutathione-S-transferase
i.p.: intraperitoneal
KO: knockout
P: postnatal day
PB: phosphate buffer
qPCR: quantitative real-time polymerase chain reaction
RGC: retinal ganglion cells
SC: superior colliculus
WT: wildtype
