# Supplementary figures and images for: Downstream mediators of Ten-m3 signalling in the developing visual pathway
Source: BMC Neurosci. 2017 Dec 6;18:78. doi: 10.1186/s12868-017-0397-5 (PMC5718065; doi:10.1186/s12868-017-0397-5)

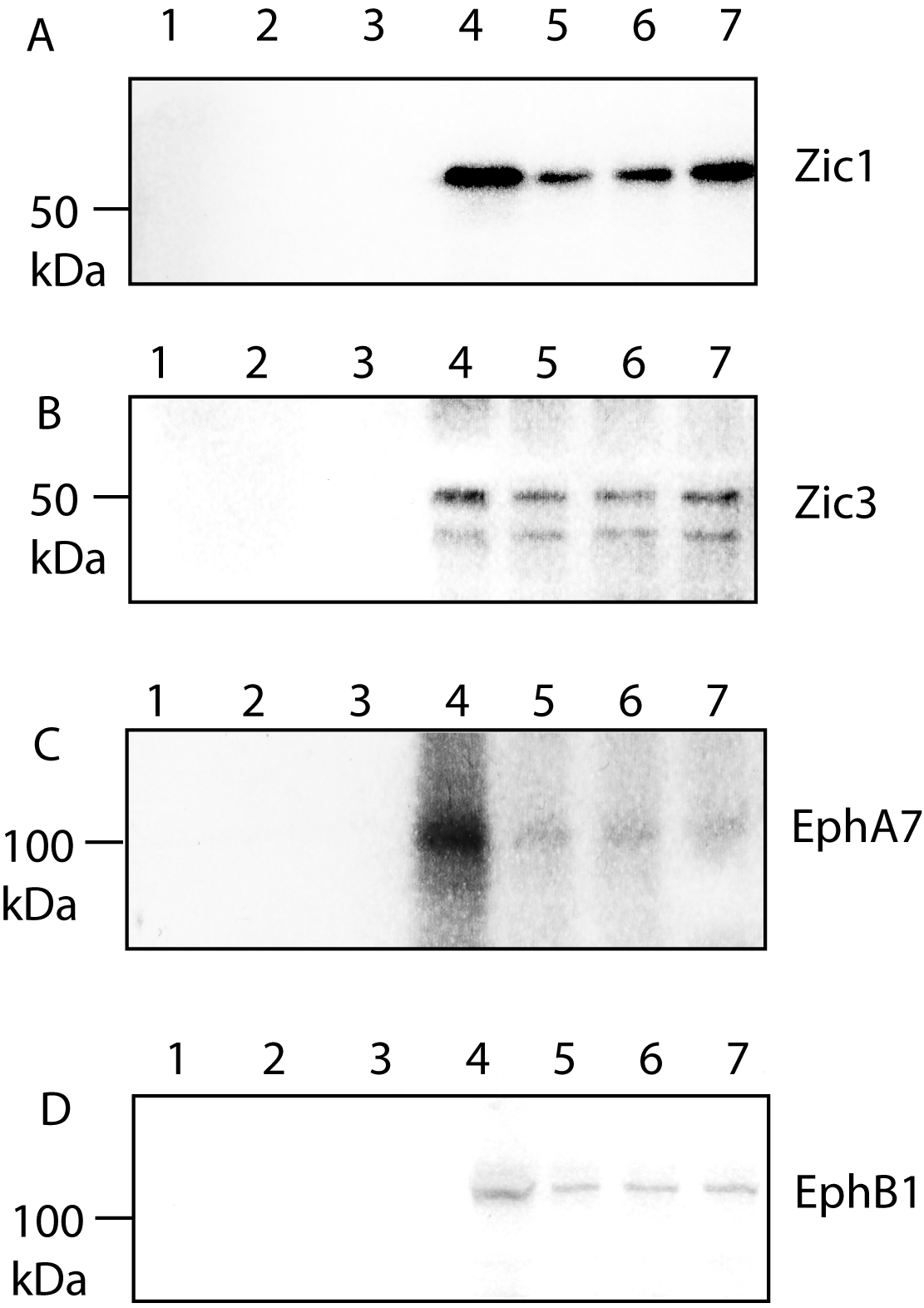

Supplement: Supplementary file 1 — Additional file 1: Fig. S1. Western blots showing immunodetection of proteins corresponding to the expected sizes of Zic1 (A), Zic3 (B), EphA7 (C) and EphB1 (D) following GST pull-down assay with mouse P1 whole brain lysates. In each case the lanes show: sepharose beads (lane 1), GST alone (lane 2), Ten-m3-GST fusion protein probe (lane 3), whole brain lysate positive control (lane 4), and supernatants of beads only (lane 5), GST alone (lane 6), and Ten-m3-GST (lane 7). The prominent bands corresponding to the expected size of each of the proteins can be observed in the whole brain lysate and supernatants of all conditions, but not in any of the pull-down lanes. Most notably, no band is present in the Ten-m3-GST pull-down lane (lane 3), suggesting that there is no direct binding interaction between the intracellular domain of Ten-m3 and any of these molecules. [file 12868_2017_397_MOESM1_ESM.tif]
